# *Strongyloides stercoralis* and Organ Transplantation

**DOI:** 10.1155/2013/549038

**Published:** 2013-05-27

**Authors:** Bhalaghuru Chokkalingam Mani, Moses Mathur, Heather Clauss, Rene Alvarez, Eman Hamad, Yoshiya Toyoda, Mark Birkenbach, Mustafa Ahmed

**Affiliations:** ^1^Section of Cardiology, Temple University School of Medicine, Philadelphia, PA 19140, USA; ^2^Section of Infectious Diseases, Temple University School of Medicine, Philadelphia, PA 19140, USA; ^3^Advanced Heart Failure & Transplantation, Temple University School of Medicine, 3401 North Broad Street, Parkinson Pavilion, 9th Floor, Philadelphia, PA 19140, USA; ^4^Section of Cardiothoracic Surgery, Temple University School of Medicine, Philadelphia, PA 19140, USA; ^5^Department of Pathology, Temple University School of Medicine, Philadelphia, PA 19140, USA

## Abstract

*Strongyloides* is a parasite that is common in tropical regions. Infection in the immunocompetent host is usually associated with mild gastrointestinal symptoms. However, in immunosuppressed individuals it has been known to cause a “hyperinfection syndrome” with fatal complications. Reactivation of latent infection and rarely transmission from donor organs in transplanted patients have been suggested as possible causes. Our case highlights the importance suspecting *Strongyloides* in transplant recipients with atypical presentations and demonstrates an incidence of donor derived infection. We also review the challenges associated with making this diagnosis.

## 1. Case

A 60-year-old Hispanic male originally from Puerto Rico with end-stage ischemic cardiomyopathy status postorthotopic heart transplantation (OHT) in July 2012 presented 2 months after transplant with fatigue and malaise. On arrival he appeared ill but afebrile. He had an episode of hemoptysis and was admitted for further evaluation. 

His posttransplant course was complicated by recurrent episodes of cellular rejection requiring both oral and intravenous pulse dose steroids. His immunosuppression regimen at time of presentation included mycophenolate mofetil 1500 mg twice daily in addition to tacrolimus 2.5 mg and 20 mg prednisolone daily. His most recent endomyocardial biopsy (EMBx) revealed resolution of cellular rejection with normal hemodynamics. On hospital day 1, he underwent repeated EMBx which was negative for evidence of cellular and antibody-mediated rejection. Echocardiography and right heart catheterization revealed normal allograft function and hemodynamics. He subsequently developed a worsening respiratory distress requiring transfer to the cardiac intensive care unit and intubation. Thereafter, he became profoundly hypotensive requiring initiation of norepinephrine in addition to broad spectrum antimicrobial coverage. 

**Figure 1:**
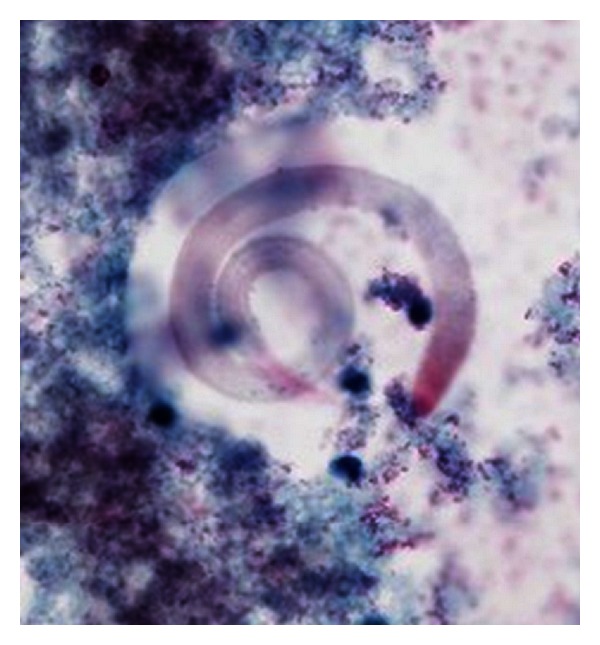
BAL specimen showing adult worm.

Over the next 72 hours, he became increasingly unstable requiring additional vasopressor support. Shortly after intubation, he underwent bronchoscopy and on day 4 of admission, bronchoalveolar lavage (BAL) revealed *Strongyloides stercoralis* as the parasite was visualized (Figure 1). Ivermectin and albendazole were initiated via nasogastric tube.

With these interventions, the patient's hemodynamic and respiratory status improved. However, his neurological status did not improve despite withdrawal of sedation. Therefore a lumbar puncture was performed which revealed vancomycin resistant enterococcus and *Strongyloides* in the cerebrospinal fluid. Linezolid and daptomycin were therefore added to his regimen, but his neurological status never recovered and life-sustaining support was withdrawn on hospital day 26. 

**Figure 2:**
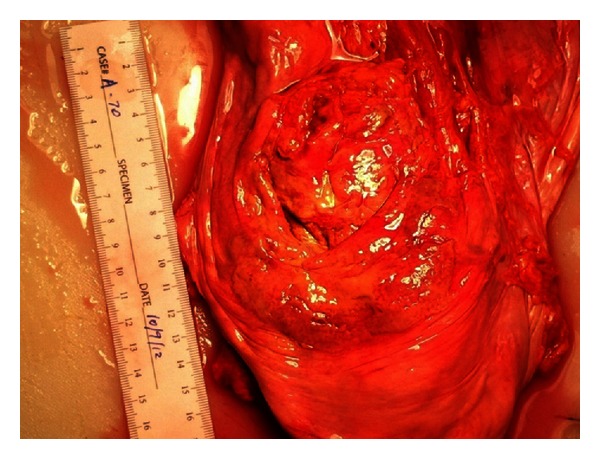
Anterior surface of the bladder dome revealing parisitoma at autopsy.

**Figure 3:**
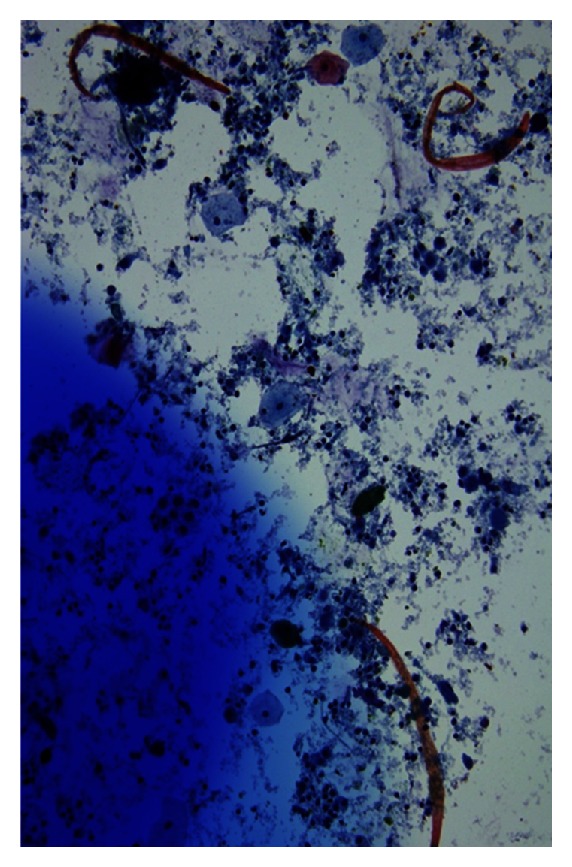
Washings from parisitoma revealing larval forms.

On autopsy, larval forms were identified in the lung, heart, lymph nodes, and liver. Additionally, a peritoneal exudate (Figure 2) was discovered on the serosal surface of the anterior rectum and bladder dome. Microscopic examination revealed this to be a peritoneal parasitoma with viable adult and larval forms (Figure 3 and see the video in Supplementary Material available online at: http://dx.doi.org/10.1155/2013/549038). Examination of the gastrointestinal tract revealed adult parasites within the jejunal bypass segment but not in the blind loop duodenum in this gentleman who had previously undergone a Roux-en-Y gastric bypass, suggesting postgastric surgery infection.

Once *Strongyloides* was identified, the Centers for Disease Control and Prevention (CDC) was contacted. Pretransplant donor serum was tested for *Strongyloides* antibody which was found to be positive while pretransplant recipient serum was compared and found to be negative (Table 1). The other institutions involved in organ transplantation from the same donor were informed of the developments by the CDC.

## 2. Discussion

*Strongyloides stercoralis* is a helminthic intestinal parasite which is endemic in tropical and subtropical regions affecting 30 to 100 million people worldwide [1]. The southeast United States is considered endemic with most cases occurring in immigrants and veterans [2]. *Strongyloides* infection occurs by penetration of larvae through the skin on exposure to contaminated soil. Upon entry, the larvae travel through the bloodstream and reach the alveolar spaces of the lungs. The larvae are then expectorated and swallowed resulting in infection of the small intestine. The larvae mature into adult worms which then mate and release eggs. These eggs produce larvae which are either excreted through feces or mature into filariform larvae which can infect the intestinal tissue or penetrate perirectal mucosa to enter the circulatory system resulting in the so-called “auto infection” [3, 4]. It is in this fashion that disseminated infection can cause bacteremia with gut flora, as demonstrated in our case. *Strongyloides* infection is frequently asymptomatic or causes minimal gastrointestinal symptoms. However, hyperinfection with larval dissemination to systemic organs can occur. Those with compromised cell-mediated immunity are at increased risk for developing hyperinfection and its complications. This includes long-term chronic steroid use, transplant recipients including bone marrow and solid organs, and patients with HIV, HTLV infection [3, 5–7].

From 1991 to 2006 nearly 400 deaths due to *Strongyloides* have been reported. There were 16 reported cases of *Strongyloides* hyperinfection between 2006 and 2010, largely in immunocompromised individuals, with an estimated mortality rate of 69%. In the transplant population, strongyloidiasis has been reported in recipients of hematopoietic stem cells, kidneys, liver, heart, intestine, and pancreas [4, 7]. The sources of infection have been identified as chronic preexisting infection in the recipient or in rare cases from transmission of infection through the donor allograft organ [8]. 

## 3. *Strongyloides* in Orthotopic Heart Transplantation

To our knowledge there are 6 reported cases of *Strongyloides* in cardiac transplant patients to date (Table 2) [8–13]. The source of infection in these patients seems to be either preexisting chronic infections in the recipient or from the allograft itself as in the case presented by Brügemann et al. All patients presented with nonspecific or vague gastrointestinal complaints. *Strongyloides* hyperinfection in this population carries a high risk of mortality as only 1 of the previously reported 6 cases survived.

## 4. Donor Derived *Strongyloides* Infection in Transplant Recipients

Donor derived *Strongyloides* infection is a rare but a reported occurrence. To our knowledge there are 10 prior cases in the literature (Table 3) [12, 14–19]. Symptoms were observed within 6 weeks to 9 months after transplantation with a wide variety of presentations including rashes and nonspecific gastrointestinal complaints, as well as fulminant hyperinfection syndrome and respiratory distress. Oral albendazole and ivermectin were used for treatment in the majority of cases. In one case, ivermectin was continued intermittently as a form of secondary prophylaxis. In another case, specific FDA approval was obtained to administer veterinary ivermectin (Ivomec 1% injection) on a compassionate-use basis. Five of the reported cases experienced successful treatment, whereas 4 patients died due to the infection and its related complications. One patient was successfully treated but died later in the same hospitalization due to acinetobacter bacteremia. In only 3 of the reported cases was the donor confirmed to have *Strongyloides*. In the remaining cases, the donor allograft was suspected as a result of clinical reasoning, which took into account the donor's origin, evidence of *Strongyloides* infection in multiple recipients from the same donor, and pathologic findings.

## 5. Diagnosis and Treatment

Various diagnostic tests are available, with a wide range of diagnostic accuracy (Table 4) [1, 20–24]. Stool culture is only useful in chronic strongyloidiasis if there is regular and constant larval output, thereby making it unreliable [2, 21, 23]. Treatment regimens include one or two doses of ivermectin and/or a 7-day course of oral albendazole. One and two doses of ivermectin at two-week intervals were more likely to attain higher parasitological cure rate compared to albendazole [25]. In *Strongyloides* hyperinfection syndrome, ivermectin 200 mcg/kg/day has been used for up to two weeks until stool tests are negative. Although it is not FDA approved, anecdotal evidence suggests that use of subcutaneous or rectal ivermectin at the same dose may be useful in cases of malabsorption or poor oral intake [4]. 

## 6. Summary

*Strongyloides* hyperinfection can happen anytime after transplant. However, there seems to be predilection to strike within the first 3 months during times of increased immunosuppression. ISHLT guidelines recommend the use of antiviral, fungal, and protozoal prophylaxis immediately after a cardiac transplant; however, this does not include prophylaxis against *Strongyloides*. While screening is recommended for those potential recipients with an appropriate travel history, there is no recommended screening program in potential donors [26, 27]. Although cost issues have to be taken into account before instituting a standard protocol for screening for such a rare occurrence, the frequency of *Strongyloides* infection may increase in the future given the changing demographics of donor and recipient pools. Screening could be narrowed only to high-risk populations such as those from endemic areas. Fitzpatrick et al. have made a case to screen transplant donors and recipients of Hispanic origin given their potential for increased exposure due to origin or travel from endemic areas [28]. Also atypical symptoms and/or signs in transplanted patients should prompt an early investigation with serological assays, BAL, or upper GI endoscopy whichever might apply to the situation. In the reported 6 heart transplant recipients who developed *Strongyloides* hyperinfection, attempted treatment was not successful in 5 patients. This highlights the importance of earlier screening in transplanted or potential transplant recipients. The arrival of luciferase precipitation systems assay and real time polymerase chain reaction testing may pave the way for a better screening tool. Our recommendation would also be to empirically treat with ivermectin or albendazole along with a reduction in immunosuppression in transplant recipients with atypical clinical presentations. We would also recommend testing of at-risk donors with treatment of the organ recipients once results become available. 

## Supplementary Material

Video of the parisitoma washings revealing viable larval forms in motion.

## Figures and Tables

**Table 1:** Allograft recipient posttransplant *Strongyloides* confirmation.

Allograft	Pretransplant stronglyoides IgG enzyme immunoassay	Post-transplant confirmatory test	Presentation	Treatment	Outcome
Heart	Negative	Bronchoscopy	Respiratory distress	Ivermectin and albendazole	Death
Liver	Negative	Not available	Sudden death	Not available	Death
Kidney	Negative	Endoscopy	Rash, fever	Ivermectin and albendazole	Recovered
Kidney/pancreas	Negative	Endoscopy	Abdominal abscess	Ivermectin and albendazole	Allograft failure

**Table 2:** Strongyloidiasis in cardiac transplantation.

Source	Allograft	Time from transplant	Risk factors	Presentation	Diagnostic test	Treatment	Outcome
Schaeffer et al. [9]	Heart	2 months	Travel to southeastern US	Perforated colon	BAL examination	Thiabendazole × 15 days Ivermectin × 15 days	Death

El Masry and O'Donnell [10]	Heart	41 days	From Kentucky	Respiratory distress	Alveolar tissue on autopsy	None	Death

Mizuno et al. [11]	Heart/ kidney	28 days	From Florida	Respiratory distress	Autopsy findings	None	Death

Roxby et al. [8]	Heart	2 months	Immigrant from Ethiopia	Dyspnea Abdominal pain Nausea	Sputum examination	Oral ivermectin	Death

Brügemann et al. [12]	Heart	6 weeks		Abdominal pain Anorexia Nausea	Skin biopsy	Oral ivermectin × 15 days Albendazole oral × 10 days	Successful treatment

Grover et al. [13]	Heart	4 weeks	From Southeastern US	Nausea vomiting	Duodenal biopsy	Ivermectin	Death

**Table 3:** 

Source	Allograft	Time from transplant	Demographic risk factor	Presenting feature	Diagnostic test	Treatment	Outcome
Said et al. [14]	Kidney	48 days	Cadaveric donor from South Asia	SHS	BAL examination	Oral and rectal albendazole/ivermectin	Death
	Kidney	90 days	Cadaveric donor from South Asia	SHS	BAL examination	Oral and rectal albendazole/ivermectin	Death
	Kidney	92 days		SHS	BAL examination	Oral and rectal albendazole/ivermectin	Death

Huston et al. [15]	Kidney	90 days	Cadaveric donor from Puerto Rico	Fever and respiratory distress	BAL examination	Oral and rectal albendazole/ivermectin Trial of veterinary ivermectin (after case-specific FDA approval) × 3 doses	Successful treatment

Hoy et al. [16]	Kidney Kidney	33 days 64 days	None	Diarrhea and fever Cough and fever	Stool analysis Urine analysis	Thiabendazole × 5 d Thiabendazole × 5 d	Death Successful treatment

Patel et al. [17]	Intestine	9 months	Donor from Honduras	Nausea/vomiting and abdominal discomfort Fevers	Small bowel and colon endoscopic biopsy BAL examination	Oral ivermectin/thiabendazole and rectal ivermectin × 10 d	Successful treatment initially but died later during the same hospitalization due to acinetobacter bacteremia

Ben-Youssef et al. [18]	Pancreas	49 days	Donor was immigrant to the USA	Hematuria and epigastric pain	Duodenal biopsy	Oral thiabendazole/ivermectin × 7 d	Successful treatment

Brügemann et al. [12]	Heart	6 weeks	Donor from Surinam	Abdominal pain and rash	Skin biopsy	Oral ivermectin × 15 d Oral albendazole × 10 d	Successful treatment

Rodriguez- Hernandez et al. [19]	Liver	2.5 months	Donor from Ecuador	Anorexia and diarrhea	Sputum and stool sample examination	Oral albendazole/ivermectin × 2 weeks, then ivermectin only × 2 weeks, followed by intermittent ivermectin secondary prophylaxis	Successful treatment

SHS: *Strongyloides* hyperinfection syndrome.

BAL: bronchoalveolar lavage.

**Table 4:** Currently available *strongyloides* diagnostic studies.

Diagnostic test	Sensitivity/specificity	Advantages	Disadvantages
Stool smear Baermann [20]	75%	Easily obtained	Requires multiple specimens to improve sensitivity and specificity

ELISA IgG [21]	97% sensitivity 95% specificity	High sensitivity High specificity	False negatives Other filarial reactions can cause false positivity Remains positive for extended periods even after treatment

Stool on Agar plate culture [1, 22]	96% sensitivity	High sensitivity	Requires at least 2 days

PCR [23, 24]	>95% sensitivity >95% specificity	High specificity Becomes negative after successful treatment	Not all diagnostic centers are equipped to perform test

Luciferous immunoprecipitation System [21]	97% sensitivity 100% specificity	<2.5 hours High sensitivity and Specificity Seroconversion after treatment	Not all labs have capability to perform test
